# Real-World Engagement With a Generative AI Conversational Agent for Mental Health Support: Retrospective Descriptive Study

**DOI:** 10.2196/95811

**Published:** 2026-06-26

**Authors:** Kelsey McAlister, Courtney Jewell, Jennifer Huberty

**Affiliations:** 1 Fit Minded, Inc, 2901 E Greenway Road PO Box, Phoenix, AZ, 30271, United States, 1 (602) 935-6986

**Keywords:** digital mental health interventions, digital therapeutics, chatbots, industry science, real-world setting, user satisfaction

## Abstract

**Background:**

Generative artificial intelligence (GenAI) conversational agents are increasingly integrated within digital mental health interventions (DMHIs). However, empirical data on real-world engagement, usage patterns, and satisfaction with GenAI conversational agents remain limited.

**Objective:**

This study examined real-world engagement among users who interacted with the GenAI conversational agent within Mental, a DMHI designed to support mental health. We aimed to (1) characterize users engaging with Mental’s GenAI conversational agent, (2) examine real-world usage patterns, (3) examine satisfaction and user feedback following sessions, and (4) explore preliminary predictors of engagement with Mental’s GenAI conversational agent.

**Methods:**

This retrospective study analyzed naturalistic user data from 5082 paid subscribers who engaged with Mental’s GenAI conversational agent between October 2024 and March 2026. Users’ onboarding characteristics (ie, sex, mindset, distress level, desire for greater discipline, and primary stressors) and session satisfaction were collected via optional app-native items; session-level engagement metrics were captured through backend app usage data. Descriptive statistics were used to characterize user demographics and usage patterns. Session satisfaction was compared across temporal engagement variables using ANOVAs and independent-samples *t* tests. As an exploratory aim, session-level mixed-effects logistic regression was used to estimate predictors of session-to-session return, with session satisfaction as the primary predictor and moderation by self-reported mindset.

**Results:**

Among users reporting onboarding data, 78.8% (2610/3312) identified as male and 90.0% (2667/2964) reported moderate-to-high distress on an app-native item. A total of 59,602 sessions were recorded (mean 11.8 sessions per user), most frequently occurring in the evening (17,206/59,602, 28.9%) and outside traditional business hours (37,181/59,602, 62.4%). Mean session satisfaction was high (mean 4.5, SD 0.9) and did not differ by time of day or day of the week. The most commonly selected session descriptors were “Insightful” (9236/19,883, 46.5%), “Felt seen” (7974/19,883, 40.1%), and “Good advice” (7510/19,883, 37.8%). The session-to-session return rate was 92.6%, and 69.4% (3528/5082) of users returned after their first session. In an exploratory analysis, session satisfaction was a significant predictor of return (odds ratio 1.35, 95% CI 1.14-1.60; *P*<.001), although this finding should be interpreted as hypothesis-generating.

**Conclusions:**

Users engaged with a GenAI conversational agent within the Mental app outside of traditional care hours and presented with high return rates. Objective behavioral engagement data (eg, session frequency, timing, and session-to-session return rate) provide novel evidence that GenAI conversational agents may sustain real-world engagement, including among individuals who face barriers to traditional mental health services. Future research should determine whether these engagement patterns translate into clinically meaningful outcomes.

## Introduction

Mental health conditions affect a substantial portion of adults in the United States, with recent estimates indicating that nearly 1 in 5 adults (approximately 23% of the adult population) experiences a mental illness in a given year [1]. Despite the high prevalence of mental illness, there is a significant unmet need for mental health care [2]. Traditional mental health care in the United States is primarily delivered through in-person, clinician-led models that, while effective, are inherently time- and resource-intensive [2-4]. Care typically depends on scheduled appointments, provider availability, and ongoing coordination, which limits how many individuals can be served at a given time [2].

As demand has grown, digital mental health interventions (DMHIs), including mobile apps, online programs, and self-guided tools, have emerged as a way to expand access and reduce strain on the system [5]. These tools offer practical advantages such as convenience, on-demand access, and lower cost [6,7]. However, real-world use has been inconsistent. Many digital tools demonstrate strong initial uptake but limited sustained engagement, with substantial dropout after early sessions and limited data on how they are used in noncontrolled, applied contexts [8-10]. As a result, important questions remain regarding which design characteristics and engagement strategies are associated with sustained naturalistic use.

Recent advances in generative artificial intelligence (GenAI) have led to rapid growth in artificial intelligence (AI)–powered conversational agents embedded within DMHIs [5,7,11]. These AI agents differ from earlier static or rule-based AI tools by enabling dynamic, free-text dialogue that can adapt in real time to user input [12]. While these tools are increasingly adopted and frequently described as scalable, on-demand supports, the empirical literature remains limited in several important ways. Much of the existing research has focused on symptom outcomes in controlled or short-term study samples, often with predominantly female participants and limited attention to real-world deployment [13]. Objective behavioral data are also limited. Few studies report when agents are used, how often users return between sessions, or how session-level satisfaction unfolds in naturalistic contexts [13-15]. Additionally, although retention challenges are widely reported across DMHIs [8,9], little is known about engagement patterns specific to AI conversational agents or how engagement varies by users’ baseline traits and demographic characteristics [15]. Taken together, these gaps limit generalizability, obscure who engages with and benefits from AI conversational agents, and hinder understanding of whether these tools are reaching intended populations and supporting sustained use in real-world settings.

One example of this emerging class of AI tools is the Mental app, a commercially available DMHI [16], geared primarily for adult men. Its core feature is a GenAI conversational agent that delivers on-demand, free-text mental health support. Unlike rule-based or single-modality DMHIs, Mental is designed to flexibly draw from multiple evidence-based therapeutic approaches (eg, cognitive behavioral therapy [CBT], acceptance and commitment therapy [ACT], and motivational interviewing), dynamically selecting techniques based on user input and conversational context. Because Mental is used outside a controlled research context, it provides an opportunity to better understand how a GenAI conversational agent is used and engaged within real-world settings to support mental health.

The purpose of this study was to examine real-world engagement among users who interacted with the GenAI conversational agent within Mental, a DMHI designed to support mental health. We aimed to (1) characterize users engaging with Mental’s GenAI conversational agent, (2) examine real-world usage patterns of the GenAI conversational agent, (3) examine satisfaction and user feedback following sessions with the GenAI conversational agent, and (4) explore preliminary predictors of engagement with the GenAI conversational agent. The primary contribution of this study is the large-scale characterization of objective behavioral engagement patterns in a real-world setting, providing an empirical foundation for future evaluations of GenAI conversational agents aimed at supporting adult mental health.

## Methods

### Study Design and Participants

This retrospective study analyzed secondary data from the Mental app, a commercially available DMHI that incorporates a GenAI conversational agent to support users’ mental health. Eligible participants were identified through app usage records using a retrospective cohort sampling approach. Participants were included in this study if they (1) were paid users of the Mental app, (2) were US residents, and (3) engaged with the platform’s GenAI conversational agent (defined as completing at least 1 session with Mental’s GenAI conversational agent with nonmissing, nonzero duration) between October 10, 2024, and March 12, 2026. Users who did not meet these criteria were not eligible for this study. No direct recruitment or prospective enrollment occurred, as all analyses were conducted using existing Mental user data. Each user was assigned a unique, deidentified study identifier to maintain confidentiality and ensure that records were not duplicated across analyses. As this was a retrospective study, the sample size was determined by the availability of complete data. This study was conducted and reported in accordance with the STROBE (Strengthening the Reporting of Observational Studies in Epidemiology) guidelines for observational cohort studies (Checklist 1).

### Ethical Considerations

The study protocol was reviewed by the Biomedical Research Alliance of New York Institutional Review Board (Study ID 26-006-1708) and was determined to qualify for exemption under US federal regulations for human subjects research involving secondary analysis of existing data. Because the study used retrospective, deidentified data and involved no direct participant contact, informed consent procedures were not required. All data were deidentified before analysis. Participants did not receive compensation for inclusion in this research.

### The Mental App

The Mental app is a commercially available DMHI, accessible for download through mobile app marketplaces (eg, Apple App Store and Google Play). Users download the app, create an account, and enroll to access its features. The Mental app is marketed primarily for adult men and emphasizes performance, discipline, and stress management through action-oriented content. The platform includes multiple tools (eg, music to support sleep or focus and structured training activities), though this study focuses specifically on user engagement with one of Mental’s core features, the GenAI conversational agent, as captured through routine app usage data.

The GenAI conversational agent is grounded in multiple empirically supported therapeutic approaches, including CBT, ACT, motivational interviewing, and other cognitive, behavioral, and skills-based strategies. While earlier DMHIs incorporating AI agents have relied on structured, rule-based, or decision-tree frameworks grounded primarily in manualized CBT [17-19], the Mental app’s GenAI conversational agent is designed to draw flexibly from multiple evidence-based therapeutic approaches based on user input and context. This design reflects the flexibility observed in routine clinical practice, where therapists adapt techniques to individual needs. The agent is built on a shared GenAI infrastructure used across related products developed by the same company (ie, The Path), enabling consistency in underlying model architecture while supporting different user experiences [20]. When engaging with the Mental app, users select a preferred AI persona (eg, differing in tone, personality, and/or coaching style) and choose a session length (eg, approximately 8, 15, or 30 min) at the start of each interaction, allowing for flexible and customizable engagement. From there, users can engage in real-time, free-text dialogue to support their mental health and well-being. The agent incorporates safety guardrails, including in-session and postsession crisis warnings, emergency escalation prompts directing users to seek care when indicated, and ongoing quality monitoring through manual review of randomly sampled sessions. Proprietary model architecture and training data details are not publicly disclosed.

### Study Measures

#### Onboarding Measures

Mental users complete baseline self-report items during onboarding, before engaging with the app’s features, including sex (male or female), mindset (indifferent/apathetic, negative/pessimistic, positive/optimistic, wise/reflective, or not sure), desire to be more disciplined (yes, no, or not sure), distress level (super, very, fairly, a little, or none), and primary stressors (relationships, work, finances, not achieving goals, family, health, parenting, loss, school, or other). All onboarding prompts were optional, and completion rates are reported. Onboarding items were custom-developed for the Mental platform and do not have established psychometric properties. In particular, the distress item is a single, app-native question with unknown reliability and construct validity, and responses should not be interpreted as clinically meaningful indicators of distress. Full item wording and response options are reported in Multimedia Appendix 1 (Table S2).

#### Session Satisfaction

Following each completed interaction with the GenAI conversational agent, users provided a brief satisfaction rating using an app-native, single-item 5-star scale ranging from 1 (lowest satisfaction) to 5 (highest satisfaction). Users were also prompted to select one or more predefined session descriptors reflecting their experience of the conversation, including “Insightful,” “Good advice,” “Felt seen,” “Empowering,” “Life-changing,” “Misunderstood me,” “Superficial,” “Confusing,” “Rigid,” or “Boring.” The satisfaction measure and session descriptors were developed for use within the platform and have not been formally validated, and their reliability, construct validity, and sensitivity to change are unknown. Submission of session ratings and descriptors was optional; thus, they are subject to response bias and do not represent all sessions.

#### GenAI Conversational Agent Engagement Metrics

Engagement metrics were derived from timestamped interaction logs capturing the use of Mental’s GenAI conversational agent. Engagement with the GenAI conversational agent was operationalized at both the user level and session level. At the user level, the total number of completed AI sessions per user was calculated to capture overall engagement. For users with ≥2 sessions, the average time between sessions was calculated as the number of days between the first and last session divided by the number of completed sessions minus 1.

At the session level, session duration was calculated as the total session duration in seconds divided by 60. Session timestamps were used to derive 3 temporal engagement variables: (1) time of day, (2) business-hour status, and (3) day type. All temporal variables were derived using the session start timestamp. Time of day was categorized into 4 mutually exclusive session-level periods: morning (5 AM-11:59 AM), afternoon (noon-4:59 PM), evening (5 PM -9:59 PM), and late night (10 PM-4:59 AM). Business-hour status was defined as sessions occurring during business hours (9 AM-4:59 PM, Monday-Friday) or after hours (all other times). Day type was classified as weekend (Saturday-Sunday) or weekday (Monday-Friday).

### Statistical Analyses

To characterize the users engaging with the GenAI conversational agent, user-level descriptive statistics were calculated using means and SDs for continuous variables and frequencies and percentages for categorical variables. Self-reported distress levels were categorized as high (very, super), moderate (fairly), and low (a little, none). Separate binary indicators were created for each primary stressor.

To examine real-world usage patterns, the proportion of sessions occurring across temporal engagement variables (ie, time of day, business hour status, and day type) was summarized. Session intervals, defined as the average number of days elapsed between consecutive sessions, were calculated for users with 2 or more sessions. Session satisfaction ratings were summarized using means and SDs and were compared across temporal engagement variables (ie, time of day, business-hour status, and day type) using a 1-way ANOVA and independent-samples *t* tests, as indicated. Differences in session duration across temporal engagement variables were evaluated using independent-samples *t* tests. Postsession descriptors were summarized at the user level (ie, the proportion of users endorsing each descriptor among those who applied at least one) and the session level (ie, the proportion of tagged sessions in which each descriptor was selected). Session-to-session return rate was calculated as the proportion of sessions followed by a subsequent session within the same user. The return rate following the first session was also calculated. Session frequency was categorized based on total sessions per user, and distributions were summarized descriptively.

As an exploratory aim, session-level mixed-effects logistic regression was used to estimate preliminary predictors of session-to-session return. Session satisfaction rating (1-5) was included as the primary predictor. Self-reported mindset was included as the main effect, with “not sure” as the reference category, and interaction terms between satisfaction and self-reported mindset were tested to assess whether the association between satisfaction and subsequent session return differed by self-reported mindset categories. Sex was included as a covariate, with female as the reference category, due to prior evidence linking sex to engagement and retention in DMHIs [21]. A random intercept for users was included to account for repeated sessions nested within individuals. Odds ratios (ORs) with 95% CIs are reported. Analyses were conducted using available data without imputation. Models were estimated using listwise deletion, and observations with missing data were excluded from the models. Because session satisfaction ratings were voluntary, the regression analytic sample was restricted to sessions with nonmissing satisfaction, mindset, and sex data, resulting in a reduced and potentially self-selected subsample. Findings from this analysis should be considered hypothesis generating rather than confirmatory. Statistical significance was defined as *P*<.05. All analyses were conducted using R (version 4.4.2) [22].

### Methods for the Mitigation of Bias

To mitigate potential conflicts of interest, the following safeguards were applied: (1) research questions were developed by the Fit Minded scientific team based on scientific merit and were not prescribed by the client; (2) study outcomes were not guaranteed as contractual deliverables; (3) the analysis plan was prespecified prior to data access; and (4) data analysis and interpretation were led by Fit Minded authors operating independently of The Path. No author’s employment status or compensation is contingent upon the direction or outcome of the findings.

## Results

### Data Availability and Descriptive Characteristics

Of the 24,792 users during the study period, 9133 were excluded for not being US residents, and 10,224 of the remaining 15,659 did not have paid subscriptions. Of the 5435 paid US users, 353 had no valid sessions (ie, sessions with nonmissing, nonzero duration) and were excluded, resulting in an analytic sample of 5082 users. Completion for optional onboarding variables ranged from 58.3% to 58.4% across items (mindset: n=2963/5082; distress: n=2964/5082; discipline: n=2967/5082; primary stressors: n=2963/5082). Completion for session-level feedback was 58.3% (34,748/59,602) for satisfaction ratings and 33.4% (19,883/59,602) for postsession descriptors. See Table S3 in Multimedia Appendix 2 for more details on missing data, including analyses comparing users with and without complete onboarding data.

Among users who completed onboarding items, the majority identified as male (2610/3312, 78.8%), reported high distress (1962/2964, 66.2%), and indicated a desire to be more disciplined (2678/2967, 90.2%) on app-native items. Indifferent (1022/2963, 34.5%) and negative (916/2963, 30.9%) were the most commonly reported mindsets. The most commonly endorsed primary stressors were relationships (1000/2883, 34.7%), followed by work (458/2883, 15.9%) and finances (407/2883, 14.1%; Table 1).

**Table 1.** Descriptive characteristics of paid users (N=5082).

Characteristic	Participants, n (%)^a^
Sex	
Male	2610 (51.4)
Female	702 (13.8)
Missing	1770 (34.8)
Mindset	
Indifferent	1022 (20.1)
Negative	916 (18)
Positive	457 (9)
Wise	144 (2.8)
Not sure	424 (8.3)
Missing	2119 (41.7)
Desire to be more disciplined	
Yes	2678 (52.7)
No	91 (1.8)
Not sure	198 (3.9)
Missing	2115 (41.6)
Self-reported distress level	
High	1962 (38.6)
Moderate	705 (13.9)
Low	297 (5.8)
Missing	2118 (41.7)
Primary specific stressors	
Relationships	1000 (19.7)
Work	458 (9)
Finances	407 (8)
Not achieving goals	337 (6.6)
Family	192 (3.8)
Health	166 (3.3)
Parenting	111 (2.2)
A loss	82 (1.6)
School	51 (1)
Other	159 (3.1)
Missing	2199 (41.7)

a Percentages reflect the proportion of the full analytic sample (N=5082).

### GenAI Conversational Agent Engagement Characteristics

A total of 59,602 sessions were recorded during the study period. Users completed an average of 11.8 (SD 42.5) sessions. Sessions most commonly occurred in the evening (5 PM-9:59 PM; n=17,206, 28.9%), followed by morning (5 AM-11:59 AM; n=16,866, 28.3%), afternoon (noon-4:59 PM; n=14,072, 23.6%), and late night (10 PM-4:59 AM; n=11,458, 19.2%). The majority of sessions occurred outside traditional business hours (n=37,181, 62.4%), compared with during business hours (9 AM-4:59 PM, Monday-Friday; n=22,421, 37.6%). Most sessions occurred on weekdays (n=43,738, 73.4%) rather than weekends (n=15,864, 26.6%).

Among users with 2 or more sessions (n=3528), the mean interval between sessions was 12.8 (SD 30.3) days. Mean session duration was 15.8 (SD 10.1; median 15.1, IQR 8.6‐20.6) minutes overall and 16.1 (SD 10.1; median 15.2, IQR 8.7‐20.9) minutes during after-hours sessions (Table 2). After-hours sessions were significantly longer than those occurring during business hours (*P*<.001), and weekend sessions (mean 16.0, SD 10.3; median 15.2, IQR 8.6‐20.1) were slightly longer than weekday sessions (mean 15.8, SD 10.0; median 15.1, IQR 8.6‐20.5; *P*=.01), although this may reflect the large sample size rather than a meaningful practical difference.

**Table 2.** App engagement descriptives.

Characteristic	Value
Completed sessions per user, mean (SD)	11.8 (42.5)
Time of day of sessions, n (%)	
Late night (10 PM-4:59 AM)	11,458 (19.2)
Evening (5 PM-9:59 PM)	17,206 (28.9)
Afternoon (12 PM-4:59 PM)	14,072 (23.6)
Morning (5 AM-11:59 AM)	16,866 (28.3)
Business vs nonbusiness hours, n (%)	
After hours	37,181 (62.4)
Business hours	22,421 (37.6)
Weekend vs weekday, n (%)	
Weekend	15,864 (26.6)
Weekday	43,738 (73.4)
Session intervals (n=3528 users with ≥2 sessions), mean (SD)	12.8 (30.3)
Session duration (min), mean (SD)	
Overall (N=59,602)	15.8 (10.1)
After-hours (n=37,181)	16.1 (10.1)
Business hours (n=22,421)	15.4 (10.1)
Weekend (n=15,864)	16.0 (10.3)
Weekday (n=43,738)	15.8 (10.0)
Session satisfaction, mean (SD)	
Overall satisfaction (n=34,748)	4.5 (0.9)
After-hours (n=21,969)	4.5 (0.9)
Business hours (n=12,779)	4.5 (0.9)
Weekend (n=9136)	4.5 (0.9)
Weekday (n=25,612)	4.5 (0.9)

The overall session-to-session return rate (ie, the proportion of sessions followed by a subsequent session) was 92.6%, and the return rate following the first session was 69.4%, although engagement varied across users. Specifically, 30.6% (1554/5082) of users completed a single session, 14.2% (723/5082) of users completed 2 sessions, 9.1% (461/5082) of users completed 3 sessions, 5.7% (287/5082) of users completed 4 sessions, 15.6% (791/5082) completed 5 to 9 sessions, and 25.0% (1266/5082) completed 10 or more sessions. See Figure S1 Multimedia Appendix 3 for a retention curve. Return rates remained high even following lower satisfaction ratings (eg, approximately 88.1% return following a rating of 1). Male users completed slightly fewer sessions on average than female users (mean 12.3 vs 12.9).

### Satisfaction With the GenAI Conversational Agent

Mean session satisfaction was high overall (mean 4.5 of 5, SD 0.9), and there were no significant differences between session satisfaction, business-hour status (*P*=.06), or day type (*P*=.59; Table 2). When selecting from predetermined descriptors to describe their experience after a session, 3086 users assigned at least 1 postsession descriptor. Among users who applied at least 1 postsession descriptor (n=3086), the most commonly endorsed descriptors were “Insightful” (n=2072, 67.1%), “Good advice” (n=1904, 61.7%), “Felt seen” (n=1761, 57.1%), “Empowering” (n=1419, 46%), and “Life-changing” (n=682, 22.1%; Table 3). At the session level, a similar pattern was observed, with these 5 descriptors ranking as the most frequently selected across all tagged sessions (n=19,883; Table 3). However, submission of session satisfaction ratings and descriptors was optional and may reflect response bias toward more satisfied users.

**Table 3.** Session descriptors summaries at the user and session levels.

Tag	Users, n (%)^a^	Sessions, n (%)^b^
Insightful	2072 (67.1)	9236 (46.5)
Good advice	1904 (61.7)	7510 (37.8)
Felt seen	1761 (57.1)	7974 (40.1)
Empowering	1419 (46.0)	5474 (27.5)
Life-changing	682 (22.1)	2532 (12.7)
Misunderstood me	505 (16.4)	951 (4.8)
Superficial	484 (15.7)	969 (4.9)
Confusing	419 (13.6)	778 (3.9)
Rigid	302 (9.8)	721 (3.6)
Boring	241 (7.8)	426 (2.1)

a Percent of users who tagged a session reflects the proportion of users who applied at least one postsession descriptor (n=3086).

b Percent of tagged sessions reflects the proportion of sessions in which at least one descriptor was applied (n=19,883) that included each descriptor. Percentages may exceed 100% as multiple descriptors could be selected per session.

### Predictors of Engagement With the GenAI Conversational Agent

As an exploratory analysis, separate logistic regression models were used to examine whether session satisfaction, mindset, sex, and satisfaction-by-mindset interactions were associated with the likelihood of returning for a subsequent session (Table 4). This analysis was conducted on a restricted sample due to listwise deletion. The regression analytic sample comprised 23,480 sessions from 2428 users, accounting for approximately 39% of the total 59,602 sessions and 48% of the total 5082 users.

**Table 4.** Session-level predictors of return for a subsequent session.^a^

Predictor	OR^b^ (95% CI)	*P* value
Session satisfaction (rating)	1.35 (1.14-1.60)	<.001
Sex (male vs female)	0.71 (0.57-0.88)	.002
Mindset		
Indifferent mindset	1.17 (0.46-2.95)	.74
Negative mindset	2.02 (0.81-5.06)	.13
Positive mindset	0.98 (0.32-2.97)	.97
Wise mindset	0.55 (0.14-2.15)	.39
Rating × mindset interactions		
Rating × Indifferent mindset	0.89 (0.73-1.09)	.27
Rating × Negative mindset	0.93 (0.76-1.13)	.46
Rating × Positive mindset	0.96 (0.75-1.22)	.73
Rating × Wise mindset	1.09 (0.81-1.48)	.57

a Due to listwise deletion across session satisfaction ratings, mindset, andsex, the regression analytic sample comprised 23,480 sessions from 2428 users (vs 59,602 sessions and 5082 users in the full sample). The reference category for sex was female. The reference category for mindset was “not sure.”

b OR: odds ratio.

Session satisfaction was significantly associated with return for a subsequent session (OR 1.35, 95% CI 1.14-1.60; *P*<.001), indicating that each 1-point increase in satisfaction rating was associated with a 35% increase in the odds of return. Mindset was not significantly associated with return (all *P*>.05), and rating-by-mindset interaction terms were also nonsignificant (all *P*>.05), suggesting that the association between satisfaction and return did not differ across mindset categories. Male sex was associated with lower odds of return relative to females (OR 0.71, 95% CI 0.57-0.88; *P*=.002). However, observed return rates were high for both males (92.9%) and females (93.3%). Given the psychometric limitations of the satisfaction measure and the self-selected nature of the analytic sample, these findings should be interpreted as preliminary and hypothesis generating.

## Discussion

### Overview

The purpose of this study was to examine real-world engagement among users who interacted with Mental’s GenAI conversational agent, a DMHI designed to support mental health. We aimed to (1) characterize users engaging with Mental’s GenAI conversational agent, (2) examine real-world usage patterns of the GenAI conversational agent, (3) examine satisfaction and user feedback following sessions with the GenAI conversational agent, and (4) explore preliminary predictors of engagement with the GenAI conversational agent. By documenting real-world usage and engagement patterns at scale, this study establishes an empirical foundation for future evaluations of GenAI aimed at supporting adult mental health. The primary contribution of this study is the objective, behavioral engagement data derived from timestamped app logs, including session frequency distributions, temporal patterns of engagement, and session-to-session return rates. Mental users were predominantly male, reported moderate-to-high levels of distress, and indicated a desire to be more disciplined on app-native items. Users identified most often as having indifferent or negative mindsets, and the most commonly reported stressors were relationships, work, and finances. On average, users completed 12 sessions with a mean interval of approximately 13 days between sessions, and most sessions occurred outside of traditional business hours and on weekdays. The session-to-session return rate was high, with 69.4% of users returning after their first session. The average session satisfaction was high, with no significant differences in session satisfaction across time of day or day of week. Mental users most frequently characterized sessions as “Insightful,” “Good advice,” and “Felt seen.” Session-to-session return rate was significantly predicted by session satisfaction, but return rates remained high even following lower satisfaction ratings, and male sex was associated with lower odds of return compared with females. However, these findings should be interpreted as hypothesis generating, given the unvalidated satisfaction measure and the self-selected analytic subsample on which it was estimated.

Of Mental users who responded to onboarding items, approximately 79% of users were male, which is not surprising considering Mental has a male-targeted marketing strategy. The majority of users reported high or moderate levels of distress and indicated a desire to become more disciplined on app-native items. Although these responses were collected via an unvalidated item and cannot be interpreted as clinically meaningful indicators of distress, the pattern suggests that males endorsing distress at onboarding are more likely to engage with mental health support when it is action-oriented (eg, skills building, goal setting), rather than delivered primarily through emotion-focused disclosure or discussion (eg, talk therapy, peer support groups; [23,24]). GenAI conversational agents may better engage populations that traditionally underutilize mental health services [25,26]. For men, in particular, private, autonomy-supported formats may reduce the self-stigma and masculinity-related barriers that often deter help-seeking behavior [27,28]. At onboarding, most users reported indifferent or negative mindsets. Although prior digital health research has linked positive affect and readiness to sustained platform engagement [29,30], the current findings suggest that neutral or negative mindsets do not prevent users from engaging with conversational AI agents. Relationships, work, and finances were the most frequently reported stressors, aligning with commonly reported stressors among US adults [31]. Future research using longitudinal and experimental designs is needed to explore whether baseline mindset and users’ primary stressors causally influence engagement trajectories with GenAI conversational agents.

Users completed approximately 12 sessions, with the majority of sessions occurring outside of traditional business hours. Specifically, sessions most commonly occurred in the evening or in the morning. The temporal patterns of engagement observed in this study are consistent with evidence suggesting that DMHIs, including mobile- and web-based platforms and text-based support apps, may provide support outside of typical service hours [32,33]. This is important considering the well-documented barriers to accessing traditional mental health support, such as scheduling constraints and costs [34]. The mean interval between sessions (~13 d) may reflect an episodic pattern of engagement, with users potentially returning to the platform at moments of perceived need rather than habitual engagement. Future research should compare engagement patterns and outcomes (eg, burnout, stress, and relationship quality) between GenAI conversational agents and those receiving traditional mental health support to evaluate differences in accessibility, utilization patterns, and overall effectiveness.

Users demonstrated strong session-to-session return rates, which is important given that AI-driven conversational agents and many DMHIs more broadly struggle to retain users past a few sessions [35,36]. High session-to-session return rates were observed across both male and female users, underscoring the ability of GenAI conversational agents (like Mental) to sustain engagement across sexes. However, male users completed fewer sessions on average than female users and had lower odds of returning. Importantly, while frequent return is commonly viewed as a positive indicator of engagement and value of DMHIs [9,36], it may not solely reflect beneficial use. In some cases, a high session return rate could reflect an overreliance on AI support rather than skill building or behavior change [10]. This interpretation is relevant considering the moderate-to-high distress reported by the users at onboarding on an app-native item, as users endorsing greater distress may be more likely to engage frequently for immediate support, which reflects a different pattern of use than skill acquisition alone. Future research should explore how therapeutic alliance may influence engagement and outcomes with GenAI conversational agents, including whether there is an optimal threshold that maximizes benefit while minimizing risks of overreliance, and should further examine additional predictors of return, such as perceived need and habit formation.

Mental users reported high satisfaction ratings after sessions, as captured by a single-item, app-native rating scale. Direct comparisons with prior work are limited, given the lack of standardized satisfaction measures across the literature. However, the ratings observed here align with, and in some cases exceed, satisfaction benchmarks reported across other AI-driven and rule-based mental health chatbots and communication agents [37,38]. Importantly, the satisfaction measure used in this study is a single-item rating without established psychometric properties, and submission was optional. Thus, satisfaction findings must be interpreted in the context of these limitations. Satisfaction rates were consistent across all temporal engagement variables (time of day, business hour status, and day type), indicating that the perceived quality of interaction did not vary by time of use. Session descriptors characterized interactions as “Insightful,” “Good advice,” and “Felt seen.” Although these descriptors were custom developed for the Mental app and have not been formally validated, the results suggest users generally perceived sessions positively. The high satisfaction rates and qualitative descriptions may be attributed to the AI’s conversational style that leverages multiple evidence-based frameworks (eg, CBT, ACT, and motivational interviewing) within a single session. Unlike rule-based, scripted chatbots, GenAI conversational agents may better reflect the adaptive, responsive quality of traditional clinical interactions, potentially addressing the rigidity that users have reported as barriers to engagement with chatbots [39,40]. Notably, a subset of users characterized sessions as “Rigid” or “Boring,” suggesting that while GenAI agents are an advancement over rule-based systems, opportunities exist to further personalize the experience for some users [41]. Comparative qualitative designs may extend these findings to identify the features of GenAI conversational agents that drive the highest user satisfaction, focusing on efficacy and perceived value instead of mere engagement.

Session satisfaction ratings were a significant predictor of return rate and remained consistent across mindsets in exploratory regression analyses. This may suggest that the relationship between session satisfaction and continued engagement with the platform was not influenced by the user’s mindset. These patterns align with evidence, suggesting that perceived interaction quality and satisfaction are critical to sustained engagement with DMHIs, including chatbot-based formats [42]. However, the results were derived from an unvalidated single-item measure and a voluntary, self-selected sample (approximately 39% of total sessions). Users who chose to submit satisfaction ratings may be more engaged or satisfied than those who did not, which may directionally inflate the relationship between satisfaction and return. As such, these findings should be treated as hypothesis generating rather than confirmatory evidence of meaningful association. Return rates remained high even following lower satisfaction ratings, which may reflect a need-based return, such that users reengage due to mental health needs and limited access to alternative support, independent of previous self-reported session quality [36]. Future research should explore whether return rates vary among users with access to alternative mental health supports versus those without.

Several aspects of this study strengthen its contributions. The primary contributions (eg, session frequency, timing distributions, and session-to-session return rates) are derived from objective, timestamped behavioral logs and are not subject to the psychometric limitations that affect self-report measures. These data were drawn from a naturalistic, publicly available DMHI rather than a controlled research setting, supporting the ecological validity of our findings. In addition, this study provides one of the first empirical investigations of user engagement with a GenAI conversational agent that is marketed toward men, a population that remains underrepresented in digital mental health research.

This study is not without limitations. First, the inclusion criteria required the sample to be paying subscribers of Mental to ensure an internally consistent sample, though this approach may have introduced selection bias and reduced the generalizability of the findings. Users who made a financial investment in Mental may be more motivated to sustain engagement and report higher satisfaction rates compared to free or trial users. Paid subscribers may also differ from the broader population of individuals with mental health needs in terms of socioeconomic status, baseline motivation, and health literacy, which may influence platform engagement. The sample is also predominantly male, which limits generalizability to other sex and gender groups. Future research should examine engagement and satisfaction among free or trial users, and across more demographically diverse samples, to determine how the patterns generalize across groups. Second, demographic and onboarding data were largely incomplete, which is common in observational digital health studies [43], but this limited our ability to characterize the user sample or examine how engagement or satisfaction rates vary across user demographic profiles. Because demographic information was not systematically collected, we were unable to assess or adjust for potential sampling biases related to age, race/ethnicity, or socioeconomic status. Future work should prioritize more complete demographic capture to enable subgroup analyses and improve generalizability.

Third, this study did not use validated outcome measures. Onboarding items and session satisfaction metrics were collected using custom items developed for the Mental app and do not have established psychometric properties. In particular, the distress onboarding item is a single, unvalidated app-native item with unknown reliability and construct validity. The characterization of users as experiencing moderate-to-high distress reflects self-reported responses on this app-native item and should not be interpreted as a clinically validated or diagnostically meaningful indicator of distress. In addition, onboarding and satisfaction ratings were optional and may reflect response bias toward more engaged or satisfied users. The exploratory regression model was further constrained by the self-selected nature of the analytic sample, such that users who submitted ratings may have differed from nonraters in ways that may directionally alter the association between satisfaction and return. This selection mechanism, compounded with the psychometric limitations of the self-report measures, means that findings from the regression model are best understood as a secondary, hypothesis-generating analysis. Future studies should integrate validated outcome measures, improve the completeness of user-reported data, and include appropriate comparison conditions to evaluate whether engagement with GenAI conversational agents supports clinically meaningful change over time. Fourth, this study was limited to users who engaged with Mental’s GenAI conversational agent, and comparative data for users who accessed other app features without engaging in the AI component were not available for this study. Future research should examine whether engagement patterns and satisfaction differ among users who engage with a GenAI conversational agent versus solely using other app features, as this would strengthen the causal inference about the AI component of the study.

### Conclusions

This retrospective study characterized real-world users who engaged with a GenAI conversational agent within the Mental app, a publicly available DMHI designed to support mental health. Users were predominantly male, reported moderate-to-high distress on an app-native item at baseline, and engaged with the platform outside of business hours. Objective behavioral engagement data (eg, session frequency, temporal patterns of engagement, and session-to-session return rates) represent the primary contribution of this study, suggesting that GenAI conversational agents can reach users in real-world settings. Session satisfaction and return rates were high, though these findings should be interpreted cautiously given the unvalidated, single-item measure and self-selected subsample on which they are based. Future research is needed to incorporate validated outcome measures to determine whether engagement patterns translate into clinically meaningful outcomes.

## Supplementary material

10.2196/95811 Multimedia Appendix 1 Onboarding items.

10.2196/95811 Multimedia Appendix 2 Missingness of data.

10.2196/95811 Multimedia Appendix 3 Retention curve.

10.2196/95811 Checklist 1 STROBE checklist.

## Abbreviations

ACT: acceptance and commitment therapy
AI: artificial intelligence
CBT: cognitive behavioral therapy
DMHI: digital mental health intervention
GenAI: generative artificial intelligence
OR: odds ratio
STROBE: Strengthening the Reporting of Observational Studies in Epidemiology
