# Supplementary material for: Real-World Engagement With a Generative AI Conversational Agent for Mental Health Support: Retrospective Descriptive Study
Source: JMIR Form Res. 2026 Jun 26;10:e95811. doi: 10.2196/95811 (PMC13308752; doi:10.2196/95811)
Supplement: Multimedia Appendix 1 [file formative-v10-e95811-s001.docx]

**Supplement Material 2. Onboarding Items**

| Table S2. User onboarding items and domains. | | |
| --- | --- | --- |
| **Domain** | **Onboarding question** | **Response Options** |
| Gender | Users selected one of two buttons at the start of their onboarding quiz. | Male |
|  |  | Female |
| Mindset | How would you describe your mindset? | Indifferent - apathetic, unmotivated, disconnected |
|  |  | Negative - angry, pessimistic, sad, low self-worth |
|  |  | Positive - optimistic, grateful, confident |
|  |  | Wise - clear mind, insightful, compassionate |
|  |  | Not Sure |
| Desire to be more disciplined | Do you want to become more disciplined? | Yes |
|  |  | No |
|  |  | Not Sure |
| Distress level | Lately, how stressed, anxious, or depressed have you been? | Super |
|  |  | Very |
|  |  | Fairly |
|  |  | A little |
|  |  | None |
| Primary stressor | What has been the biggest source of stress for you recently? | Relationships |
|  |  | Work |
|  |  | Finances |
|  |  | Not achieving goals |
|  |  | Family |
|  |  | Health |
|  |  | Parenting |
|  |  | A loss |
|  |  | School |
|  |  | Other |
| *Users were only able to select one response for each onboarding item. | | |
