# Supplementary material for: Real-World Engagement With a Generative AI Conversational Agent for Mental Health Support: Retrospective Descriptive Study
Source: JMIR Form Res. 2026 Jun 26;10:e95811. doi: 10.2196/95811 (PMC13308752; doi:10.2196/95811)
Supplement: Multimedia Appendix 2 [file formative-v10-e95811-s002.docx]

**Supplement 3. Missingness of Data**

Among user-level variables, approximately 58% of users provided data on mindset, emotional distress, desire for discipline, and primary stressor, and 65.2% providing gender information (Table S3). Missingness for these variables ranged from 34.8% to 41.7%. At the session level, satisfaction ratings were available for 58.3% of sessions (34,748 of 59,602), while post-session descriptors were provided for 33.4% of sessions (19,883/59,602).

Sessions with satisfaction ratings were longer on average than those without ratings (mean = 18.0 vs. 12.7 minutes; p < .001), and a small but statistically significant difference was observed in the proportion of after-hours sessions (63.2% vs. 61.2%; p < .001).

Users with and without onboarding data did not significantly differ in number of sessions completed or proportion of after-hours sessions across mindset, emotional distress, desire for discipline, and primary stressor variables (all p > .05). Specifically, tests comparing users with versus without data showed no significant differences in sessions completed (p = .083–.088) or after-hours use (p = .279–.300). Overall, these findings suggest that missingness was more strongly associated with session-level engagement than with availability of user-level onboarding data.

| Table S3. Missingness of data. | | | | | | |
| --- | --- | --- | --- | --- | --- | --- |
| Variable | Level | Total N | Non-missing N | Missing N | % Complete | % Missing |
| Gender | User-level | 5,082 | 3,312 | 1770 | 65.2 | 34.8 |
| Mindset | User-level | 5,082 | 2,963 | 2,119 | 58.3 | 41.7 |
| Emotional distress | User-level | 5,082 | 2,964 | 2,118 | 58.3 | 41.7 |
| Desire for discipline | User-level | 5,082 | 2,967 | 2,115 | 58.4 | 41.6 |
| Primary stressor | User-level | 5,082 | 2,963 | 2,119 | 58.3 | 41.7 |
| Session satisfaction (rating) | Session-level | 59,602 | 34,748 | 24,854 | 58.3 | 41.7 |
| Session descriptors | Session-level | 59,602 | 19,883 | 39,719 | 33.4 | 66.6 |
