# Supplementary material for: Real-World Engagement With a Generative AI Conversational Agent for Mental Health Support: Retrospective Descriptive Study
Source: JMIR Form Res. 2026 Jun 26;10:e95811. doi: 10.2196/95811 (PMC13308752; doi:10.2196/95811)
Supplement: Multimedia Appendix 3 [file formative-v10-e95811-s003.docx]

**Supplement 4. Retention Curve**

User retention decreased progressively across sessions, with the largest drop-off occurring following the initial sessions. A substantial proportion of users did not return after their first session, followed by a more gradual decline in retention among those who continued engagement.

| **Figure S1. Retention Curve** |
| --- |
| **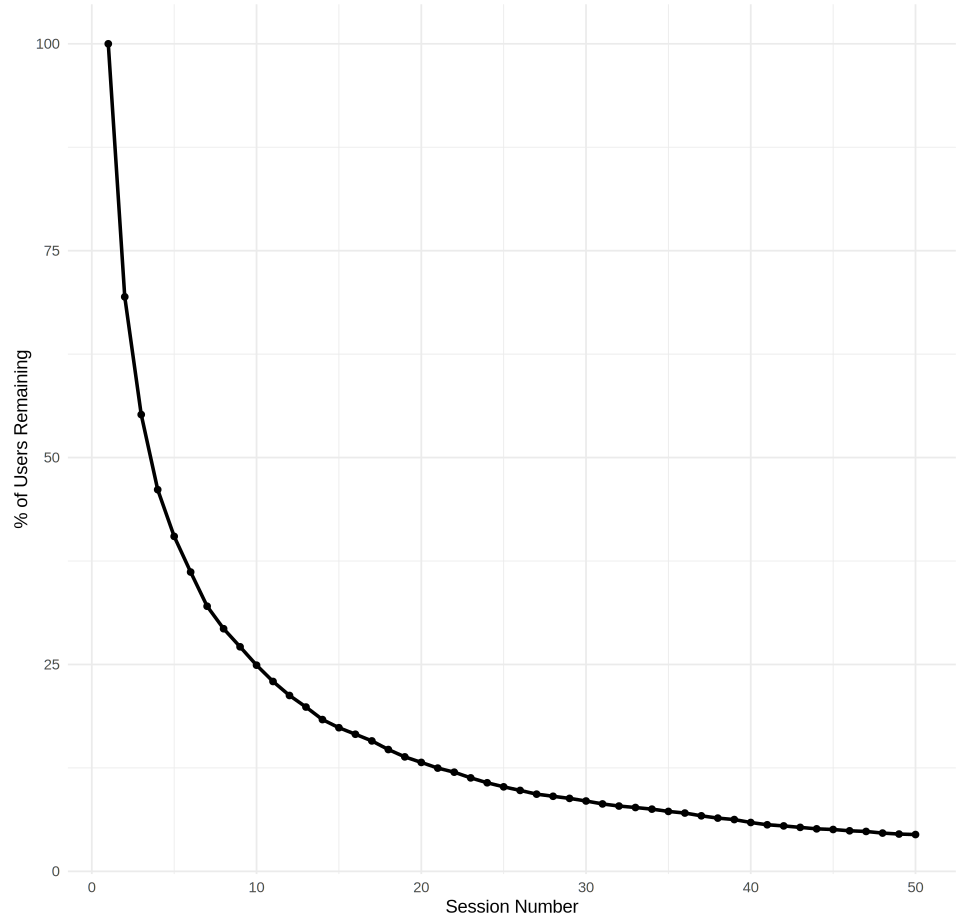** |
